# Hepatitis B Patients' Adherence to Treatment in Relation to Knowledge, Attitudes, and Practices (KAP) in the West Bank, Palestine, 2022–2023

**DOI:** 10.1111/jvh.14055

**Published:** 2024-12-23

**Authors:** Ayham Sawalmeh, Emily White Johansson, Danis Kostas, Dia'a Hjaijeh

**Affiliations:** ^1^ Preventive Medicine Department Palestinian Ministry of Health Nablus Palestine; ^2^ Mediterranean and Black Sea Programme in Intervention Epidemiology Training (MediPIET) European Centre for Disease Prevention and Control (ECDC) Stockholm Sweden; ^3^ European Centre for Disease Prevention and Control (ECDC) Stockholm Sweden; ^4^ Preventive Medicine Department Palestinian Ministry of Health Ramallah Palestine

**Keywords:** communicable diseases, cross‐sectional study, health knowledge, attitudes, and practices, hepatitis, public health

## Abstract

Hepatitis B is an infectious disease that inflicts high health and economic costs on the healthcare system. Poor adherence to treatment increases that cost. We aimed to assess the levels of knowledge, attitudes and practices (KAP) among patients in the West Bank, Palestine, and identify factors associated with good adherence. We conducted a cross‐sectional study surveying hepatitis B patients visiting primary healthcare during October 2022 until June 2023 using an interviewer‐administered questionnaire covering qualitative and quantitative aspects regarding hepatitis B. We considered adherence as good if participants received > 90% of their monthly prescription antiviral doses. Among 386 participants, the median age was 45 years (range 20–81); 80% had good adherence to treatment. Mean knowledge score was 11.4 (on a 13‐point scale), mean attitude score was 3.4 (on a 4‐point scale), mean practices score was 6 (on a 7‐point scale) and the mean overall KAP score was 21.8 (on a 24‐point scale). KAP components (Cronbach alpha = 0.820) were correlated with good adherence (*p* < 0.001). After adjustment for other factors, participants with good KAP scores had better adherence to treatment than those without (prevalence ratio: 1.41, 95% CI: 1.10–1.84, *p*‐value = 0.011). We recommend investment in education and awareness campaigns to improve adherence.

## 1 Introduction

Hepatitis B is a viral infection caused by Hepadnavirus, and it may result in acute or chronic infection. WHO estimated that 254 million people were living with hepatitis B infection in 2023, with 1.2 million new infections each year, with the hepatitis B resulting in an estimated one million deaths [1]. Acute infection can cause acute liver failure in 1%–4% of adults infected with hepatitis B virus [2]. Chronic infection can cause hepatocellular carcinoma and cirrhosis. The cumulative incidence of cirrhosis increases with the hepatitis B virus‐DNA levels and ranges from 4.5% to 36% with increasing hepatitis B viral load [3]. These sequelae pose a heavy burden on both the patients' health in the short term and long term and on the healthcare system with high costs and technical complications, thus making it a public health concern [4, 5]. Poor health practices can result in further spread of the disease and less favourable outcomes, which warrants further study [6]. In 2024, WHO released new guidelines on the prevention, diagnosis and treatment of chronic hepatitis B (HBV) infection [7]. These guidelines provide a substantial simplification and expansion of eligibility for treatment to overcome barriers in access to HBV testing and treatment, setting actions and targets to eliminate viral hepatitis by 2030 by driving new infections and deaths down to half a million each globally—a reduction of 90% and 65%, respectively. For people with chronic hepatitis B infection, antiviral treatment is highly effective, can improve survival and reduce progression of liver disease and the development of liver cancer. However, major testing and treatment gaps remain [8].

Hepatitis B in the West Bank had a chronic prevalence ranging from 2% to 6% which represents intermediate endemicity (2%–8%). In 2022, 20 deaths were recorded due to hepatitis B, resulting in 0.7 per 100,000 deaths that year, according to the Palestinian Ministry of Health annual report: the vaccination coverage exceeded 94% among newborns in 2022 [8, 9]. In Palestine, the Preventive Medicine department provides healthcare services for hepatitis patients, including counselling, laboratory investigations, follow‐up, treatment and referral if needed. Non‐adherence to treatment for chronic viral hepatitis increases the risk of virological treatment failure [10]. To mitigate this risk, it is important to identify and address factors associated with good adherence.

The study aimed to assess the level of knowledge, attitudes and practices (KAP) on hepatitis B among patients in the West Bank and to identify factors associated with good adherence to hepatitis B treatments and the reasons for poor adherence.

## 2 Materials and Methods

### 2.1 Study Design

We conducted a cross‐sectional study among hepatitis B cases under treatment and follow‐up as outpatients at the preventive medicine departments in the West Bank from October 2022 until June 2023.

### 2.2 Study Setting

Approximately 3 million Palestinians live in the West Bank, which is divided into 1.2 million in the northern districts (Nablus, Jenin, Tulkarem, Qalqilya, Salfit, Tubas), 1.1 million in the central districts (East Jerusalem, Ramallah, Bethlehem, and Jericho) and 800,000 in the southern districts (Hebron and Yatta) [11]. In Palestine, the Preventive Medicine Department provides healthcare services for hepatitis patients, including counselling, laboratory investigations, follow‐up, treatment and referral if needed. There are 14 Preventive Medicine Departments, with one in each district and three in Hebron, mostly for logistical reasons. Each department has access to the district healthcare laboratory with diagnostic capacity, although viral load detection via DNA by PCR for Hepatitis B is only available currently in the central healthcare laboratory in Ramallah.

### 2.3 Study Population and Sampling

We used simple random sampling to select hepatitis B cases ≥ 18 years old from the national registry of hepatitis B patients followed up during October 2022 until June 2023 as outpatients in the preventive medicine departments across the West Bank districts (Nablus, Tulkarem, Qalqilya, Ramallah, Bethlehem, Hebron and Yatta). We excluded individuals below 18 years of age and individuals who did not sign the consent form.

### 2.4 Sample Size

Assuming a population size in the West Bank of 3,000,000, a 50% frequency of good adherence, a precision of 5% and a design effect of 1, a sample size of 384 individuals was required.

To identify factors associated with adherence, a sample size of 206 individuals was required to detect a prevalence ratio of 1.7 with a 95% confidence interval (95% CI) and 80% power if 30% of individuals with poor KAP scores had good adherence and a ratio of unexposed to exposed individuals of 1. We calculated the sample size with OpenEPI [12].

### 2.5 Definitions

We defined hepatitis B chronic cases as individuals with a (i) positive serological tests for HBsAg and/or HBeAg twice 6 months apart, HBeAg twice 6 months apart, or either of those two together or (ii) with anti HBc total or (iii) with a positive viral load of Hepatitis B DNA by PCR [13]. We considered adherence to be good (main outcome) if participants reported missing < 3 doses in the previous month, thus making 90% of the prescribed doses per month our cut‐off point for good adherence.

The main outcome of ‘good adherence’ was defined as participant reports of missing < 3 doses in the previous month; thus, the threshold for ‘good adherence’ in our study is adhering to 90% of prescribed doses in the previous month.

The main exposure variable was the KAP score, which is a composite score including (i) ‘good knowledge’ defined as 10+ (out of 13) correct answers, (ii) ‘positive attitude’ defined as reported 3+ (out of 4) positive attitudes and (iii) ‘good practice’ as reported 6+ (out of 7) good practices. We excluded adherence from the practices score since this was our main outcome. We defined the overall KAP score as ‘good’ if each of the three composite scores achieved the criteria for ‘good’, or reaching the threshold of 75% appropriate responses (20+ of 24 questions).

### 2.6 Data Collection

Healthcare professionals (doctors/nurses) at the preventive medicine departments in the West Bank interviewed participants using a structured paper questionnaire covering information on demographics, socio‐economic status, KAP and health information about the treatment and their adherence to it, sources for information on hepatitis B and where the diagnosis was made and communicated to them (Appendix S1). The questionnaire was developed in English and translated to Arabic with pilot testing of the translated questionnaire with five potentially eligible respondents to evaluate question flow, translation and their understandings before initiating formal data collection. We conducted the interviews in Arabic for greater accessibility among the target patient group. The primary investigator performed a double data entry to minimise errors.

### 2.7 Data Analysis

We calculated descriptive statistics for the study population by key demographic characteristics. To test for internal consistency of variables in each composite score (knowledge, attitude, practice), Cronbach's alpha was used to estimate a coefficient for item inter relatedness in their contribution to the overall composite score measure [14]. To identify factors associated with the binary outcome of ‘good adherence’, we calculated and used log‐binomial regression models to estimate prevalence ratios (PR) and 95% confidence intervals and adjusted PRs (aPR) using log‐binomial regression. We determined the covariates to include in the final adjusted model by including all variables with a *p*‐value < 0.25 from the univariable analysis. The final adjusted model included the following covariates: KAP score as a binary (good vs. bad), age as a categorical variable for age groups (below 33 years, 34–59 and 60 or above), gender as (female or male), education (less than high school, and high school or above), income (low income vs. high income) and side effects (had side effects from antiretroviral drugs, and suffered no side effects from anti‐retroviral drugs). The level of statistical significance was set to 0.05. We performed the analyses using R statistical software [15]. As for the multivariate model, we added all the measures of association in our model and then removed them one by one until we got the result with the best fit according to the Wald test and Akaike information Criterium (AIC).

### 2.8 Ethical Considerations

Ethical approval was obtained for this study from the scientific research review board of the primary healthcare and general health directorate, Palestinian Ministry of Health (2175‐112; 22 October 2022). Informed consent was obtained from all participants prior to their involvement in the study.

## 3 Results

### 3.1 Demographic Characteristics

Among 420 selected individuals, 386 (92%) completed the survey. The mean age of participants was 47 years with a (standard deviation (SD) = 11) years. The median age of participants was 45 (range 20–81) years. Females accounted for 42% of all respondents; 88% of participants had finished secondary education or higher (Table 1). With regards to monthly income levels, 8% of participants did not answer the question, 10% received the legally mandated minimum wage or below, which is 1800 Israeli Shekels (ILS) or less per month, the equivalent of 470$ [16]; 51% earned between 1800 and 3000 ILS, 26% between 3001 and 5000 ILS and only 5% earned more than 5000 ILS per month. The most common source of information for the hepatitis B cases was healthcare providers (62%), followed by social media and Internet (32%). Of all respondents, 37% were from the northern region, 31% from the central regions and 31% from the southern regions (Table 1).

**TABLE 1:** Characteristics of participants in the hepatitis B knowledge, attitudes and practices (KAP) survey, West Bank, 2022–2023.

	Characteristic	Number	Percent
Age (years)	18–32	23	6
	33–59	297	77
	60+	66	17
Gender	Male	224	58
	Female	162	42
Highest education level (completed)	Primary	45	11
	Secondary	186	48
	University and higher	155	41
Income in ILS^a^	1–3000	236	61
	More than 3000	118	31
	Not reported	32	8.3
Source of health information	Healthcare providers	240	62
	Social media/internet	127	33
	Educational system	9	2.3
	Family/friends	7	1.8
	TV/radio	3	0.7
Provinces	Northern region	141	37
	Central region	121	31
	Southern region	124	31
Medications for hepatitis B	Tenofovir	145	38
	Lamivudine	57	15
	Entecavir	34	9
	Lamivudine + adefovir	16	4
	Adefovir	3	1
	Not on any medications	128	33

^a^ 1USD = 3.75 ILS as of 10/10/2023.

### 3.2 Clinical Characteristics

Of all participants, 55% received the diagnosis of hepatitis B via the preventive medicine department, and the remaining from their blood bank/hospital. The mean duration of illness was 10 (SD = 7.3) years. Of the participants on treatment (*n* = 258), 56% were on Tenofovir, 22% were on Lamivudine, 13% were on Entecavir, 6% were on a combination of Lamivudine and Adefovir and 1% were on Adefovir. Of all those participants on treatment, 88% had no noticeable side effects to treatment, 10% reported that the side effects were mild and tolerable, 1% reported that the side effects were severe enough to make them consider stopping the medications and 1% complained of having side effects severe enough to cause them to stop taking the medications.

Of those 258 participants on treatment, 80% (*n* = 207) had good adherence (missed < 3 doses the previous month) and of all participants that missed any doses (*n* = 151), 66% simply forgot to take the medication, 22% reported unavailability of the drug at that time in the healthcare centre, 7% reported difficulties reaching the healthcare facility to get the medication and 5% intentionally avoided it due to the side effects.

Taking only people with poor adherence into account (*n* = 51), 45% answered that it was due to forgetfulness, 33% due to unavailability of the drug in the healthcare centre, 12% said it was the inconvenience of reaching the location of the healthcare centre and 10% said it was due to side effects suffered from the treatment. None of the participants reported the price of treatment as a reason for missing a dose. Of those 258 participants on treatment, 80% (*n* = 207) had good adherence (missed < 3 doses the previous month) and of all participants that missed any doses (*n* = 151), 66% simply forgot to take the medication, 22% reported unavailability of the drug at that time in the healthcare centre, 7% reported difficulties reaching the healthcare facility to get the medication and 5% intentionally avoided it due to the side effects.

Taking only people with poor adherence into account (*n* = 51), 45% answered that it was due to forgetfulness, 33% due to unavailability of the drug in the healthcare centre, 12% said it was the inconvenience of reaching the location of the healthcare centre and 10% said it was due to side effects suffered from the treatment. None of the participants reported the price of treatment as a reason for missing a dose.

### 3.3 Knowledge Attitudes and Practices

Among all participants, 84% had good knowledge scores (10+ correct out of 13 components), with the mean knowledge score for hepatitis B being 11.4 (SD = 1.7). Of all 386 participants, only 2 had taken part in an educational programme regarding hepatitis B. All participants correctly answered the question about the organ affected by hepatitis B. The question answered correctly by the least participants (74%) was regarding hepatitis B treatment causing addiction (Table 2).

**TABLE 2:** Score results for the hepatitis B knowledge, attitudes and practices (KAP) survey, West Bank, 2022–2023.

	Question	Participants with correct response	
		Number	Percentage
Knowledge	Hepatitis B causative agent	345	90
	Hepatitis B routes of transmission	372	96
	Hepatitis B can affect any age	327	85
	Hepatitis B primarily affects which organ	386	100
	Hepatitis B causing jaundice	355	92
	Hepatitis B treatment results in complete cure	262	68
	Hepatitis B causing cancer	312	80
	Hepatitis B patients can benefit from dietary habits	326	84
	Hepatitis B has a vaccine	366	95
	Hepatitis B needs follow‐up	382	99
	Asymptomatic Hepatitis B	322	83
	Hepatitis B medication can cause addiction	286	74
	Hepatitis B medication duration	350	91
Attitudes	Hepatitis B vaccine mandatory	344	89
	Hepatitis B tests are too many	308	80
	Hepatitis B is something that concerns me	296	77
	Willing to read and learn more about hepatitis B	376	97
Practices	Follow‐up frequency of twice a year or more	328	85
	Discussing the hepatitis B diagnosis with anyone	375	97
	Screening spouse/family against hepatitis B	371	96
	Vaccinating spouse/family against hepatitis B	340	88
	Having a healthy diet	323	84
	Frequency of abdominal ultrasound	300	78
	Good reasons to delay ultrasound a year or more	336	87
Main outcome	Hepatitis B medication adherence	207	80

Among participants, 84% (*n* = 326) had a positive attitude score of 3+ out of 4, with the mean attitude score being 3.4 (SD = 0.86). Of all participants, 76% (*n* = 293) had a good practices score of 6+ out of 7, with the mean practice score being 6.1 (SD = 1.45). The most frequent good practice was discussing the condition with someone and screening the rest of the family (96%), while the least frequent good practice was routine abdominal ultrasound follow‐ups (78%) (Table 2).

The internal consistency coefficient, Cronbach's alpha = 0.820, suggested good reliability for the measured characteristics [14]. The correlations between the KAP components were also significant (*p*‐values < 0.001), with Spearman's rank correlation coefficients exceeding 0.5 for all correlations between KAP, suggesting a significant consistency in responses between the sets of components [17] (Table 3).

**TABLE 3:** Correlation between the components in the KAP composite measures in the hepatitis B knowledge, attitudes and practices (KAP) survey, West Bank, 2022–2023.

Correlation between	Correlation coefficient	*p*
Knowledge‐attitude	0.500	< 0.001
Knowledge‐practice	0.542	< 0.001
Attitude‐Practice	0.515	< 0.001

Of all participants, 78% (*n* = 301) had a good total KAP score of 20+ out of 24, with the mean total KAP score being 20.9 out of 24 (SD = 3.5). Of participants with a good total KAP score (*n* = 301), 93% had a level of education of completing secondary school or higher, whereas 70% of those with poor KAP (*n* = 85) had this same level of education.

In the univariable analysis, the prevalence of good adherence to hepatitis B treatment among participants with an overall good KAP score was 43% higher (PR: 1.43, 95% CI: 1.11–1.86, *p*‐value < 0.007) compared with those with a poor score (Table 4). The prevalence of good adherence to treatment was also higher among those with good knowledge scores (PR: 2.02, 95% CI: 1.40–3.04, *p* < 0.001), positive attitudes towards hepatitis B treatment (PR: 1.68, 95% CI: 1.20–2.46, *p*‐value < 0.004) and good practices regarding hepatitis B (PR: 1.55, 95% CI: 1.17–2.08, *p*‐value < 0.002). Those that did not suffer side effects from antivirals were more likely to have adherence to treatments (PR: 1.32; 95% CI: 1.01–1.77; *p*‐value < 0.05) than those reporting side effects (Table 4). Adherence did not vary significantly across different types of anti‐viral treatment for hepatitis B (PR: 1.8; *p*‐value = 0.7; 95% CI: 0.91–1.0). Adherence to treatment did not significantly decrease with increasing duration of infection (PR = 0.9 for every year increase in duration of infection; 95% CI = (0.95–1.03); *p*‐value = 0.6).

**TABLE 4:** Determinants of hepatitis B treatment adherence among patients by selected characteristics, West Bank, 2022–2023.

Characteristic	Category	Adhered to treatment		Crude prevalence ratios	95% CI	*p*	Adjusted prevalence ratios	95% CI	*p*
		Number	%						
Age	18–32	19	83	Ref			Ref		
	33–59	261	88	1.06	0.69–1.75	0.795	0.98	0.63–1.63	0.944
	60+	55	83	1.01	0.61–1.74	0.974	0.95	0.57–1.65	0.851
Gender	Male	195	88	Ref			Ref		
	Female	140	87	0.99	0.80–1.23	0.947	1.01	0.81–1.26	0.908
Education	Not completed secondary	35	78	Ref			Ref		
	Secondary or higher	300	88	1.13	0.81–1.63	0.490	1.00	0.70–1.47	0.982
Income (in ILS)	Low income < 3000	205	88	Ref			Ref		
	High income > 3000	130	87	0.99	0.80–1.24	0.984	1.00	0.80–1.25	0.996
Total KAP score	Good total KAP score	273	95	1.43	1.11–1.86	0.007	1.41	1.10–1.85	0.011
	Poor total KAP score	62	66	Ref			Ref		
Side effects from antiviral treatment	Had side effects	59	75	Ref			Ref		
	Did not	276	92	1.32	1.01–1.77	0.052	1.28	0.97–1.71	0.086

In the final multivariable log‐binomial regression model, only a good total KAP score remained significant after adjusting for age, gender, education, income and side effects, indicating that those with a good KAP score were more likely to adhere to hepatitis B treatment than those with poor KAP scores (aPR: 1.41, 95% CI: 1.10–1.84, *p*‐value = 0.011) (Table 4). After adjusting for the other factors, the prevalence of good adherence to treatment was also significantly higher among those with good knowledge scores (aPR: 1.61, 95% CI: 0.97–2.76, *p* = 0.049), but positive attitudes towards hepatitis B treatment (aPR: 1.18, 95% CI: 0.97–2.76, *p*‐value = 0.473) and good practices regarding hepatitis B (aPR: 1.16, 95% CI: 0.77–1.87, *p*‐value < 0.403) did not remain significantly associated with adherence (full model not shown).

## 4 Discussion

Our study identified a significant association between overall KAP scores and good adherence to hepatitis B treatment. Good knowledge was also significantly associated with adherence in the adjusted analysis. Those findings are consistent with those reported in similar studies [18, 19, 20].

We also found that the main reason reported by participants for having missed a dose was forgetfulness, and this finding was consistent with previous studies that reported forgetfulness as the main reason for poor adherence to treatment for chronic diseases or anti‐viral treatments [21, 22]. The second main reason reported by participants for having missed a dose was unavailability of the drug in the healthcare centre (22%). This proportion rose to 33% in the group with poor adherence. Despite that, forgetfulness remained the main cause, accounting for 45% of those who missed three or more doses in the previous month. This might be explained by desirability bias, as participants might prefer to report something outside of their control as the reason for poor adherence.

Participants with better levels of education had higher KAP scores and higher adherence to medication. This was also reported in previous studies [23]. However, this apparent association of education level and good adherence did not reach the level of statistical significance in our study. Our results suggest that a person's KAP related to treatment may be a more important indicator of adherence than their overall educational level. This again points to the importance of targeted educational campaigns and promotion programmes as previously discussed.

We found no significant association between adherence and the other possible barriers, such as the duration of illness, the difficulty to reach the location of healthcare providers or the price of drugs at the healthcare provider.

We are not aware of a standard cut‐off point for adherence to hepatitis B treatment. Many studies have chosen various adherence values ranging from 80% to 95% [24, 25, 26, 27]. We chose 90% as our cut‐off point for good adherence based on the two studies [28, 29] working with entecavir, and tenofovir which collectively represent around 70% of the medication used by our hepatitis B patients. As for our KAP scores, we chose a score higher than 75% to be representative of a ‘good’ score for each of the KAP components.

### 4.1 Limitations

Our results should be interpreted after considering a few limitations. Firstly, desirability bias may have been introduced, as respondents might be more likely to report what they deemed as socially desirable behaviour in the primary healthcare setting. This may have led to an overestimation of good behaviour. To mitigate this, we informed participants that their answers were confidential and anonymous and that refusal to participate in the study would not impact their standing with regards to receiving treatment from the preventive medicine department or anything else. In addition, quantitative studies sometimes do not measure socio‐cultural and unobservable factors that might influence adherence to treatment as well as a qualitative study would. Thus, a qualitative study in the form of a focus group, for example might shed more light on factors that we may have missed.

Secondly, recall bias may have also occurred as we depended on our participants' own recollection of their adherence for the previous month. Previous studies have suggested that self‐reported adherence might be overinflated [25], whether simply as an issue of desirability or due to forgetfulness.

Thirdly, selection bias may have been introduced since severely symptomatic people may have been less likely to participate. However, we used simple random sampling to select cases. Also, to ensure a more representative sample of the study population, we included in the study participants from several provinces and districts of the West Bank. However, we could not include participants from the Gaza Strip for logistical, security and political reasons.

Another limitation included having few observations in some of our reference categories, and that could have been a barrier to obtaining significant results for some of our exposure variables. Having a larger sample size might have enabled us to detect other significant differences and associations between groups.

## 5 Conclusions

Our study among hepatitis B patients in the West Bank identified significant associations between poor adherence to treatment of hepatitis B and overall KAP after adjustment for other factors. Findings highlight the importance of initiating health education and promotion programmes to improve adherence to hepatitis B treatment by patients. Importantly, only 2 participants had previously participated in an educational campaign on hepatitis B, thus reinforcing the need for an increasing focus on hepatitis B in different media outlets, especially considering that ‘internet/social media’ were the second largest source of information for our participants, below healthcare professionals. Thus, a large portion of hepatitis B patients could benefit from such a media campaign, improving their knowledge and awareness and subsequently their adherence to treatment regimens. To improve the effectiveness of future outreach campaigns, we also recommend supplementing further work with qualitative studies to understand how best to target messages to improve adherence. As healthcare professionals were the most common source of information for hepatitis B patients, we also recommend performing surveys targeting healthcare practitioners to shed some light on any misconceptions regarding hepatitis B transmission and vaccination to help improve the KAP of the population patients and contribute towards regional and global elimination of hepatitis B.

## Author Contributions

Ayham Sawalmeh, Emily White Johansson, Danis Kostas, Dia'a Hjaijeh: conceptualisation. Ayham Sawalmeh, Danis Kostas: methodology. Ayham Sawalmeh, Dia'a Hjaijeh, Danis Kostas: software. Emily White Johansson, Ayham Sawalmeh: validation. Ayham Sawalmeh, Danis Kostas, Emily White Johansson: formal analysis. Ayham Sawalmeh, Emily White Johansson: investigation. Ayham Sawalmeh, Emily White Johansson: resources. Dia'a Hjaijeh, Ayham Sawalmeh: data curation. Ayham Sawalmeh: writing – original draft preparation. Ayham Sawalmeh, Dia'a Hjaijeh, Danis Kostas: writing – review and editing. Ayham Sawalmeh, Dia'a Hjaijeh, Emily White Johansson: visualisation. Danis Kostas, Dia'a Hjaijeh, Ayham Sawalmeh: supervision. Dia'a Hjaijeh, Emily White Johansson, Danis Kostas: project administration. Emily White Johansson, Danis Kostas and Dia'a Hjaijeh. All authors have read and agreed to the published version of the manuscript.

## Ethics Statement

The Hepatitis B KAP study in the West Bank, Palestine, 2022–2023 was approved by the IRB and deputy assistant for family and public health on 24/8/2022 and signed by the acting director of preventive medicine in primary care, Dr. Samer Al‐Asaad (code 2175‐112) on 26/10/2022. The study was conducted in accordance with the Declaration of Helsinki. No human, animal or biological samples were involved in the study, and after satisfying the data protection regulations no further approvals were required or needed.

## Consent

Informed consent was obtained from all subjects involved in the study and attached is a copy of the consent form in English and translated to Arabic.

## Conflicts of Interest

The authors declare no conflicts of interest.

## Supporting information


Appendix S1

## Data Availability

The data that support the findings of this study are available from the corresponding author upon reasonable request.

## References

[1] World Health Organization , “Global Hepatitis Report 2024: Action for Access in Low‐ and Middle‐Income Countries [Internet],” www.who.int, https://www.who.int/publications/i/item/9789240091672.

[2] T. J. Liang , “Hepatitis B: The Virus and Disease,” Hepatology 49, no. 5 Suppl (2009): S13–S21. 19399811 10.1002/hep.22881 PMC2809016

[3] U. H. Iloeje , H. I. Yang , J. Su , et al., Predicting Cirrhosis Risk Based on the Level of Circulating Hepatitis B Viral Load (Bethesda, MD: American Gastroenterological Association, 2005). 10.1053/j.gastro.2005.11.016 16530509

[4] R. Moreno and M. Berenguer , “Post‐Liver Transplantation Medical Complications,” Annals of Hepatology 5, no. 2 (2006): 77–85. 16807513

[5] Palestinian Ministry of Health Service Purchase Unit , “Referral Protocol 6: Liver Transplantation Select Cardiac Conditions [Internet],” accessed September 23, 2023, https://pdf.usaid.gov/pdf_docs/PA00WCVN.pdf.

[6] CDC , Hepatitis B Questions and Answers for the Public [Internet] (Atlanta, GA: Centers for Disease Control and Prevention, 2019), https://www.cdc.gov/hepatitis/hbv/bfaq.htm.

[7] World Health Organization , “WHO Publishes New Guidelines on Hepatitis B [Internet],” www.who.int, https://www.who.int/news/item/29‐03‐2024‐who‐publishes‐updated‐guidelines‐on‐hepatitis‐b.

[8] F. I. Lieveld , L. G. van Vlerken , P. D. Siersema , and K. J. van Erpecum , “Patient Adherence to Antiviral Treatment for Chronic Hepatitis B and C: A Systematic Review,” Annals of Hepatology 12, no. 3 (2013): 380–391. 23619254

[9] Z. Nazzal and I. Sobuh , “Risk Factors of Hepatitis B Transmission in Northern Palestine: A Case—Control Study,” BMC Research Notes 7 (2014): 190, 10.1186/1756-0500-7-190. 24678920 PMC3986636

[10] Palestinian Ministry of Health , “Palestinian Ministry of Health Annual Report 2022 Hepatitis B Deaths in the West Bank [Internet],” https://site.moh.ps/index/Books/BookType/2/Language/ar.

[11] PCBS , “Estimated Population in Palestine Mid‐Year by Governorate, 1997–2021 [Internet],” 2021, Pcbs.gov.ps, https://www.pcbs.gov.ps/Portals/_Rainbow/Documents.

[12] OpenEpi Menu [Internet] , “Openepi.com,” 2013, https://www.openepi.com/Menu/OE_Menu.htm.

[13] Centers for Disease Control and Prevention , Hepatitis‐B Information [Internet] (Atlanta, GA: Centers for Disease Control and Prevention, 2019), https://www.cdc.gov/hepatitis/hbv/.

[14] L. J. Cronbach , “Coefficient Alpha and the Internal Structure of Tests,” Psychometrika 16 (1951): 297–334, 10.1007/BF02310555.

[15] Version 2023.06.01, R Core Team , R: A Language and Environment for Statistical Computing (Vienna, Austria: R Foundation for Statistical Computing, 2013).

[16] PCBS , “The Results of the Labour Force Survey Fourth Quarter (October–December 2022) Round [Internet],” accessed September 23, 2023, www.pcbs.gov.ps, https://www.pcbs.gov.ps/post.aspx?lang=en&ItemID=4419.

[17] Y. H. Chan , “Biostatistics 104: Correlational Analysis,” Singapore Medical Journal 44, no. 12 (2003): 614–619. 14770254

[18] Y. Lailulo , M. Kitenge , S. Jaffer , O. Aluko , and P. S. Nyasulu , “Factors Associated With Antiretroviral Treatment Failure Among People Living With HIV on Antiretroviral Therapy in Resource‐Poor Settings: A Systematic Review and Metaanalysis,” Systematic Reviews 9, no. 1 (2020): 292, 10.1186/s13643-020-01524-1. 33308294 PMC7733304

[19] S. Krishnakumar , Y. Govindarajulu , U. Vishwanath , V. R. Nagasubramanian , and T. Palani , “Impact of Patient Education on KAP, Medication Adherence and Therapeutic Outcomes of Metformin Versus Insulin Therapy in Patients With Gestational Diabetes: A Hospital Based Pilot Study in South India,” Diabetes and Metabolic Syndrome: Clinical Research and Reviews 14, no. 5 (2020): 1379–1383. 10.1016/j.dsx.2020.07.026 32755838

[20] A. Nonogaki , H. Heang , S. Yi , et al., “Factors Associated With Medication Adherence Among People With Diabetes Mellitus in Poor Urban Areas of Cambodia: A Cross‐Sectional Study,” PLoS One 14, no. 11 (2019): e0225000. 31743349 10.1371/journal.pone.0225000 PMC6863566

[21] T. Barfod , H. Sørensen , H. Nielsen , L. Rodkjær , and N. Obel , “‘Simply Forgot’ Is the Most Frequently Stated Reason for Missed Doses of HAART Irrespective of Degree of Adherence,” HIV Medicine 7 (2006): 285–290, 10.1111/j.1468-1293.2006.00387.x. 16945072

[22] B. Jimmy and J. Jose , “Patient Medication Adherence: Measures in Daily Practice,” Oman Medical Journal 26, no. 3 (2011): 155–159, 10.5001/omj.2011.38. 22043406 PMC3191684

[23] F. A. Diaz‐Quijano , R. A. Martínez‐Vega , A. J. Rodriguez‐Morales , R. A. Rojas‐Calero , M. L. Luna‐González , and R. G. Díaz‐Quijano , “Association Between the Level of Education and Knowledge, Attitudes, and Practices Regarding Dengue in the Caribbean Region of Colombia,” BMC Public Health 18, no. 1 (2018): 143, 10.1186/s12889-018-5055-z. 29338712 PMC5771071

[24] N. Allard , A. Dev , J. Dwyer , G. Srivatsa , A. Thompson , and B. Cowie , “Factors Associated With Poor Adherence to Antiviral Treatment for Hepatitis B,” Journal of Viral Hepatitis 24, no. 1 (2016): 53. 27502689 10.1111/jvh.12582

[25] W. Chotiyaputta , C. Hongthanakorn , K. Oberhelman , R. J. Fontana , T. Licari , and A. S. Lok , “Adherence to Nucleos(t)ide Analogues for Chronic Hepatitis B in Clinical Practice and Correlation With Virological Breakthroughs,” Journal of Viral Hepatitis 19, no. 3 (2012): 205–212. 22329375 10.1111/j.1365-2893.2011.01494.x

[26] T. Berg , P. Marcellin , F. Zoulim , et al., “Tenofovir Is Effective Alone or With Emtricitabine in Adefovir‐Treated Patients With Chronic‐Hepatitis B Virus Infection,” Gastroenterology 139, no. 4 (2010): 1207–1217. 20600025 10.1053/j.gastro.2010.06.053

[27] L. G. van Vlerken , P. Arends , F. I. Lieveld , et al., “Real Life Adherence of Chronic Hepatitis B Patients to Entecavir Treatment,” Digestive and Liver Disease 47, no. 7 (2015): 577–583. 25936691 10.1016/j.dld.2015.03.024

[28] N. L. Allard , J. H. MacLachlan , A. Dev , et al., “Adherence in Chronic Hepatitis B: Associations Between Medication Possession Ratio and Adverse Viral Outcomes,” BMC Gastroenterology 20 (2020): 140, 10.1186/s12876-020-01219-w. 32381025 PMC7203797

[29] H. Kamezaki , T. Kanda , M. Arai , et al., “Adherence to Medication Is a More Important Contributor to Viral Breakthrough in Chronic Hepatitis B Patients Treated With Entecavir Than in Those With Lamivudine,” International Journal of Medical Sciences 10, no. 5 (2013): 567–574, 10.7150/ijms.5795. 23533048 PMC3607242

