# Supplementary material for: Hepatitis B Patients' Adherence to Treatment in Relation to Knowledge, Attitudes, and Practices (KAP) in the West Bank, Palestine, 2022–2023
Source: J Viral Hepat. 2024 Dec 23;32(1):e14055. doi: 10.1111/jvh.14055 (PMC11667092; doi:10.1111/jvh.14055)
Supplement: Supplementary file 1 — Appendix S1 [file JVH-32-0-s001.docx]

Informed consent form

Consent form You are invited to take part in filling this survey for KAP with regards to hepatitis B being performed by Dr. Ayham Sawalmeh of the Nablus preventive medicine department to assess links between KAP scores and Adherence to treatment. The survey is based on an interview conducted questionnaire and it should take no longer than 5-10 minutes. You are not required or obliged to participate under any circumstances, refusing to participate will not prejudice you or affect your treatment, follow up or management in any way shape or form, you are also free to stop at any time. The study will have no financial benefit to you directly but it will help inform the researchers to better understand the factors affecting the adherence to management and treatment, your survey answers will be confidential and no personal information that can be traced to you will be kept or dissiminated , your answers will be completely anonymous and will only be used in the aggregate for the study. Proceeding with the questionnaire entails your consent to move forward

Signature:

**KAP for Hepatitis B Survey Questionnaire**

| **Name: ID of participant:   Age: Gender: Marital status: (Single/Married/Widower/Divorced)  Profession:  Monthly Income: (1-1800/1801-3000/3001-5000-5000+)   Town/village: Place of Residence: (Refugee-Housing/Rent/Property)  Education: (Illiterate-Elementary-Secondary- University-PostGraduate)   Medical insurance: (Public-Private-None)  Name of Interviewer:   Date of interview: / /** |
| --- |

***Section 1 – Knowledge***


1- Hepatitis B is caused by a:

Virus Bacteria Fungus Stress Other


2- Hepatitis B transmission/You can get Hepatitis B from: (you can choose more than 1)

A- Blood transfusion or via contaminated syringes
B- Contaminated foods and drinks
C- Contaminated dentists equipment
D- As an Airborne Illness
E- Direct contact with an infected individual
F- Hair-Dresser/Barber from contaminated blades or scissors
G- From mother during childbirth
H- I don’t know

3- Hepatitis B can affect people of all ages

Yes No I don’t know


4- Hepatitis B damages mainly which Organ

Heart Lungs Liver Kidney Other


5- Which of these signs could suggest an infection with Hepatitis B

Cough Jaundice Dizziness Fever Dyspnea


6- The treatment for Hepatitis B can cure the condition completely

Yes No I don’t know


7- Hepatitis B can cause cancer

Yes No I don’t know


8- Diet can be important as part of Hepatitis B management

Yes No I don’t know


9- What is a good way of Hepatitis B prevention? (wait for an answer then give choices)

A- Using disposable shaving equipment
B- Eating lots of fruits and vegetables
C- Vaccination against Hepatitis B
D- Wearing face masks in public transportation
E- Others ……


10- Regular follow up is needed for Hepatitis B patients

Yes No I don’t know


11- Hepatitis B carriers can have no symptoms

Yes No I don’t know


12- The treatment for Hepatitis B can cause addiction

Yes No I don’t know


13- The medication for Hepatitis B is taken lifelong

Yes No I don’t know

 ***Section 2 - Attitudes***1- Do you think Hepatitis B vaccination should be mandatory?

Strongly Agree, Agree, I don’t know, Disagree, Strongly Disagree


2- Do you think the tests and screening the doctors ask you to do for Hepatitis B are too much?

Strongly Agree, Agree, I don’t know, Disagree, Strongly Disagree


3- Hepatitis B virus is something I find concerning

Strongly Agree, Agree, I don’t know, Disagree, Strongly Disagree


4- I would be willing to read and learn more about Hepatitis B

Strongly Agree, Agree, I don’t know, Disagree, Strongly Disagree

***Section 3 - Practices***1- How did you find out you had Hepatitis B?

A- General practitioner
B- Preventive Medicine Department
C- Hospital told you after doing test following an admission
D- Blood Bank after donating blood contacted you
E- Family member told you
F- Other….. (Please specify)


2- How long ago have you been diagnosed with Hepatitis B?

…..

3- How often do you follow up to do regular checkups and/or laboratory investigations with your local healthcare facility or with the preventive medicine department?

every 1-2 months
every 3-6 months
every 7-12 months
it has been more than a year since your last visit


4- Once you learned you had Hepatitis B, who did you discuss it with?

Spouse
Family
Friend/Co-worker
Healthcare Professional
Nobody


5- Have you ever participated in any educational program on Hepatitis B?

Yes No I don’t know

6- If yes to the previous question, can you name that program?

……………..

7- Did you recommend Hepatitis B screening for the rest of your family after being diagnosed with Hepatitis B?

Yes No I don’t know


8- Have you gotten your family vaccinated against Hepatitis B?

Yes No I don’t know


9- How would you describe your diet?

A- Lots of fruits and vegetables
B- High in fatty foods and full fat dairy/dairy products
C- High in protein from red meats
D- whole-grains rich diet
E- Other……(Please specify)


10- How often do you get an abdominal ultrasound as part of your liver screening for hepatitis B

A- Every 6 months
B- Every 6 months- 1 year
C- It has been more than a year since you done it
D- Never taken an ultrasound


11- If the answer was not A-B then why?

A- Too busy
B- No one said it was important
C- Too expensive
D- Other …… (Please specify)

12- What drug do you take for Hepatitis B?

A- I don’t take any medications
B- Lamivudine
C- Adefovir
D- Lamivudine+Adefovir
E- Entecavir
F- Tenofavir
G- I Don’t know


13- Regarding the drug schedule for your Hepatitis B medication, how would you describe your adherence to the medication schedule at any given month:

A- I never missed a dose
B- I missed no more than 3 doses
C- I missed more than 3 doses
D- I don’t really take it regularly

***If the above answer was A-B then go to the last question***


14- in case you miss doses of the drug, what would you say the reason is?

A- Side effects
B- Inconvenience of going all the way to the healthcare facility to get it
C- Forgetfullness
D- Cost of the drug
E- Unavailability of the drug
F- Others……. (Please Specify)

If the answer is not A then go to the last question

15- Which Side Effects have you experienced if any from taking your Hepatitis B medication

A- Headache
B- Muscle and Joint Pain
C- Abdominal Pain
D- Diarrhea
E- Nausea
F- Itching and Skin irritation
G- Other ……. (Please specify)

16- These Side effects or adverse events are:

A- Enough to make you stop the drug
B- Enough to make you consider stopping the drug
C- They are annoying but manageable
D- Very rarely happen/rarely Noticeable

17- What is your most trusted source when it comes to information about this subject?

TV/Radio
Social Media/Internet
Family/Friends
Educational system
Healthcare providers
Other …… (Please specify)


Please feel free to add any comments or suggestions below

…………………………


**Thank you for your participation**

#### 
